# ImmuneScore of eight-gene signature predicts prognosis and survival in patients with endometrial cancer

**DOI:** 10.3389/fonc.2023.1097015

**Published:** 2023-03-03

**Authors:** Jiahui Gu, Zihao Wang, B. O. Wang, Xiaoxin Ma

**Affiliations:** Department of Obstetrics and Gynecology, Shengjing Hospital of China Medical University, Shenyang, Liaoning, China

**Keywords:** endometrial cancer, the cancer genome atlas, immunescore, prognosis, survival

## Abstract

**Background:**

Endometrial cancer (EC) is a common gynecological cancer worldwide and the sixth most common female malignant tumor. A large number of studies conducted through database mining have identified many biomarkers that may be related to survival and prognosis. However, the predictive ability of single-gene biomarkers is not sufficiently accurate. In recent years, tumors have been shown to interact closely with their microenvironment, and tumor-infiltrating immune cells in the tumor microenvironment were associated with therapeutic effects. Furthermore, sequencing technology has evolved and allowed the identification of genetic signatures that may improve prediction results. The purpose of this research was to discover the Cancer Genome Atlas (TCGA) data to evaluate new genetic features that can predict the prognosis of EC.

**Methods:**

mRNA expression profiling was analyzed in patients with EC identified in the TCGA database (n = 530). Differentially expressed genes at different stages of EC were screened using the immune cell enrichment score (ImmuneScore). Univariate and multivariate Cox regression analyses was applied to evaluate genes significantly related to overall survival and establish the prognostic risk parameter formula. Kaplan–Meier survival curves and the logarithmic rank method were applied to verify the importance of risk parameters for the prognostic forecast. The accuracy of survival prediction was confirmed using the nomogram and Receiver Operating Characteristic (ROC) curve analysis. The mRNA expression of eight genes were measured by qRT-PCR. According to COX and HR values, NBAT1, a representative gene among 8 genes, was selected for CCK-8 assay, colony formation assay and transwell invasion assay to verify the effect on survival.

**Results:**

Eight related genes (*NBAT1*, *GFRA4*, *PTPRT*, *DLX4*, *RANBP3L*, *UNQ6494*, *KLRB1*, and *PRAC1*) were discovered to be significantly associated with the overall survival rate. According to these eight-gene signatures, 530 patients with EC were assigned to high- and low-risk subgroups. The prognostic capability of the eight-gene signature was not influenced by other elements.

**Conclusions:**

Eight related gene markers were identified using ImmuneScore, which could predict prognosis and survival in patients with EC. These findings provide a basis for understanding the application of biological information in tumors and identifying the poor prognosis of EC.

## 1 Introduction

In recent years, due to the extension of life expectancy and increase in the overall prevalence of obesity and metabolic syndrome, the incidence and mortality of endometrial cancer (EC) compared to other cancers have been continuously increasing (1). There were 382,069 new estimated cases and 89,929 estimated deaths caused by this disease in 2018 (2). EC usually occurs in postmenopausal women and only about 4% of patients are under 40 years of age (3). It is predicted that by 2025, the number of new cases and deaths will increase by 20.3% and 17.4%, respectively (2). Although most patients can be diagnosed early, some are already in the advanced stage of the disease at the time of diagnosis. Moreover, patients at identical stages can also show different responses to the same treatment and different prognoses. The mortality of EC is directly related to the poor prognostic factors that drive tumor recurrence (4). Therefore, the discovery of effective biomarkers is important to assess prognosis and identify patients at high risk for EC.

An increasing number of studies have shown the significance of tumor microenvironment (TME) in tumor progression. Synergistic interaction between cancer cells and their support cells contributes to the malignant phenotype of cancer, for example, continuous diffusion, anti-apoptosis, and evasion of immune surveillance. Therefore, TME has a significant impact on the treatment effect and clinical outcomes in cancer patients (5, 6). The main structural parts of TME are permanent stromal cells and recruited immune cells. However, the role of stromal cells in tumor angiogenesis and extracellular matrix remodeling is not completely understood (7). Some research has concentrated on the influence of immune cells in the TME on tumor growth and spread. An increasing number of studies have shown that tumor-infiltrating immune cells (TICs) in the TME are promising indicators of therapeutic effects (8).

With the advancement of high-throughput sequencing technology, investigators have set up genome databases of many diseases to understand genomic changes more systematically and clearly. Through database mining, scientists have found several biomarkers that may be related to prognosis in patients with cancer (9, 10). However, the predictive ability of single-gene biomarkers is still not sufficiently accurate. Studies have shown that evaluating genetic characteristics involving multiple genes can improve prognosis (11, 12). The polygenic prognostic characteristics of primary tumor biopsies have a guiding role in treatment. There are reports wherein the impact of multigene signatures in EC has been studied to assess prognosis and identify potential patients at high risk for EC (13, 14).

To identify biomarkers, differential gene expression analysis usually involves comparing expression levels in genes between groups and focusing on the genes whose expression levels are significantly regulated. As an emerging method, ImmuneScore can determine the difference in survival rate in patients with EC between different disease stages and finally obtain the best gene combination. This is important for tumor prognosis and survival assessment (15).

We identified new genetic characteristics that predict the prognosis of EC. We explored the Cancer Genome Atlas (TCGA) data and selected the relevant genes using ImmuneScore. Furthermore, we applied mRNA expression data from TCGA to survey and draw marker genomes in 530 patients with EC. We identified 99 mRNAs significantly related to immune cells and established a risk profile of eight genes to effectively predict the prognosis in EC patients. The risk factors obtained through ImmuneScore can independently assess the prognosis in high-risk patients and identify and verify new genetic features and biomarkers.

## 2 Materials and methods

### 2.1 Patient clinical data and mRNA expression dataset

We collected clinical data and mRNA expression profiles of EC patients from TCGA (https://cancergenome.nih.gov/) (16). The research included clinical data from 530 patients with the following parameters: matching age, stage, grade, radiation therapy, neoplasm cancer status, residual tumor, body mass index (BMI), percentage of tumor invasion, new events, and peritoneal wash (**Table 1**).

**Table 1:** Clinical pathological parameters of patients with Endometrioid cancer in this study.

Clinical pathological parameters	N (n=Excluded due to patients with missing information)	%
Age		
≥66	262	49.6
<66	266 (2)	50.4
Neoplasm cancer status		
With tumor	76	15.4
Tumor free	418 (36)	84.6
Residual tumor		
No	365	83.0
Yes	75 (90)	17.0
Stage		
I-II	381	71.9
III-IV	149 (0)	28.1
New event		
No	470	88.7
Yes	60 (0)	11.3
Grade		
G1-2	216	40.8
G3-4	314 (0)	59.2
Radiation therapy		
No	483	91.1
Yes	47 (0)	8.9
BMI		
≥28	340	68.0
<28	160 (30)	32.0
Percent tumor invasion		
No	414	90.6
Yes	43 (73)	9.4
Peritoneal wash		
With tumor	57	14.2
Tumor free	344 (129)	85.8

Inclusion Criteria: 1.Diagnosis of endometrioid carcinoma.

2.Complete clinical baseline information.

Exclusion Criteria: Exclude patients with missing clinical information.

### 2.2 Immune cell enrichment score (ImmuneScore)

We used ImmuneScore to evaluate the difference in survival rates among the EC patient groups at different stages, and thereafter assigned them as high- and low-risk groups. Used the ESTIMATE package to calculate the ImmuneScore(proportion of immune component), StromalScore(proportion of stromal component) and ESTIMATEScore(sum of the above two scores) of the EC samples. The higher the score, Represented the higher proportion of the corresponding components (immune, stromal, and tumor purity) in TME. Then, We used the edgeR algorithm (http://bioconductor.org) for preliminary screening to generate differentially expressed genes. EdgeR is a bioconductor software package used to examine the differential expression of replicated count data. Next, we used the least absolute shrinkage and selection operator (LASSO) model to select statistically significant prognostic markers from the differentially expressed genes. We analyzed the expression standards of 24,991 mRNAs in EC specimens and neighboring non-cancerous tissues. Last, we used the normalized P-value (*P <* 0.05) to determine the function for subsequent analyses.

### 2.3 Data screening and risk-parameter calculation

Log2 transformation was applied to normalize the expression profile of each mRNA. Univariate Cox regression analysis was applied to determine genes correlated with overall survival (OS), and multivariate Cox regression analysis was applied to identify genes associated with prognosis and obtain their coefficients. The selected mRNAs were then assigned as risk type (hazard ratio, HR > 1) and protective type (0 < HR < 1). By linear combination of the filtered gene expression value (weighted by its coefficient), we constructed the following hazard parameter formula: hazard parameter = ∑ (βn × gene n expression). Applying the median hazard parameter as a cut-off value, 530 patients were assigned to high- and low-risk subgroups.

### 2.4 Quantitative real-time -PCR samples and patients

A total of 20 EC tissues and 20 normal endometrial tissues were obtained from patients in the Department of Gynecology and Obstetrics of Shengjing Hospital affiliated with the China Medical University. Normal tissues were taken from patients who underwent hysterectomy for unrelated diseases of the endometrium. All patients gave informed consent. This study was approved by the Ethics Committee of Shengjing Hospital affiliated with the China Medical University. The histological diagnosis and staging were evaluated by experienced pathologists according to the International Federation of Gynecology and Obstetrics (FIGO) 2009 staging system. None of the patients received systemic treatment before surgery. Data for endometrial cancer patients are presented in **Supplementary Table S1**.

### 2.5 RNA extraction and qRT-PCR

TRIzol reagent (Vazyme, Nanjing, China) was used to extract total RNA from the tissue. PrimeScript RT-polymerase (Vazyme) was used for reverse transcription to obtain cDNAs corresponding to the target mRNAs. qRT-PCR was performed using SYBR-Green Premix (Vazyme) and specific PCR primers (Sangon Biotech Co., Ltd, Shanghai, China). Glyceraldehyde-3-phosphate dehydrogenase (GAPDH) was used as an internal control. Primer sequences are shown in **Supplementary Table S2**. The 2^−ΔΔCt^ method was used to calculate the relative fold-changes in mRNA expression.

### 2.6 Transfection of cells

SiRNA sequences targeting NBAT1, and their respective negative control (NC) counterparts were purchased from GenePharma (Shanghai, China). According to the manufacturer’s instructions, Lipofectamine 3000 (Invitrogen) was used to transfect cells with siRNA for the following experiments. Sequences of siRNA are listed in **Supplementary Table S3**.

### 2.7 Cell culture

Ishikawa cells and HEC-1A cells were cultured with RPMI 1640 (Gibco, Carlsbad, CA, USA). A 10% fetal bovine serum (FBS) (Gibco) and 1% penicillin–streptomycin was added to the medium of the cells. All cells were cultured in a humidified incubator at 37°C with 5% CO2.

### 2.8 Colony formation assay

To explore the effects of NBAT1 expression on cell proliferation, cells (1000/well) transfected with NC-siRNA or siRNA, and without si-RNA as a blank(-) group were added to each well of 6-well culture plates and incubated for two weeks. Cells were stained with 0.1% crystal violet. Finally, the number of colonies was counted by light microscopy.

### 2.9 CCK−8 assay

Ishikawa cells and HEC-1A cells were seeded in 96-well plates, CCK-8 reagent (10 µL) (Dojindo, Japan) was added to each well, and then incubated at 37°C with 5% CO2 for 3 h. The microplate reader was used to measure OD450 values of eachwell at 0h, 24h, 48h, and 72h after treatment.

### 2.10 Cell invasion assay

Transwell chambers (Corning, NY, USA) with a pore size of 8μm were used to detect cell invasion. Cells were placed into the upper chamber with 200μl serum-free medium and the chambers were precoated with Matrigel solution (BD, Franklin Lakes, NJ, USA). The lower chamber contained 10%FBS medium. After incubation for 24h,invaded cells on the lower membrane surface were fixed with 4% paraformaldehyde and stained with 0.1% crystal violet.

### 2.11 Statistical analysis

**Figure 1:**
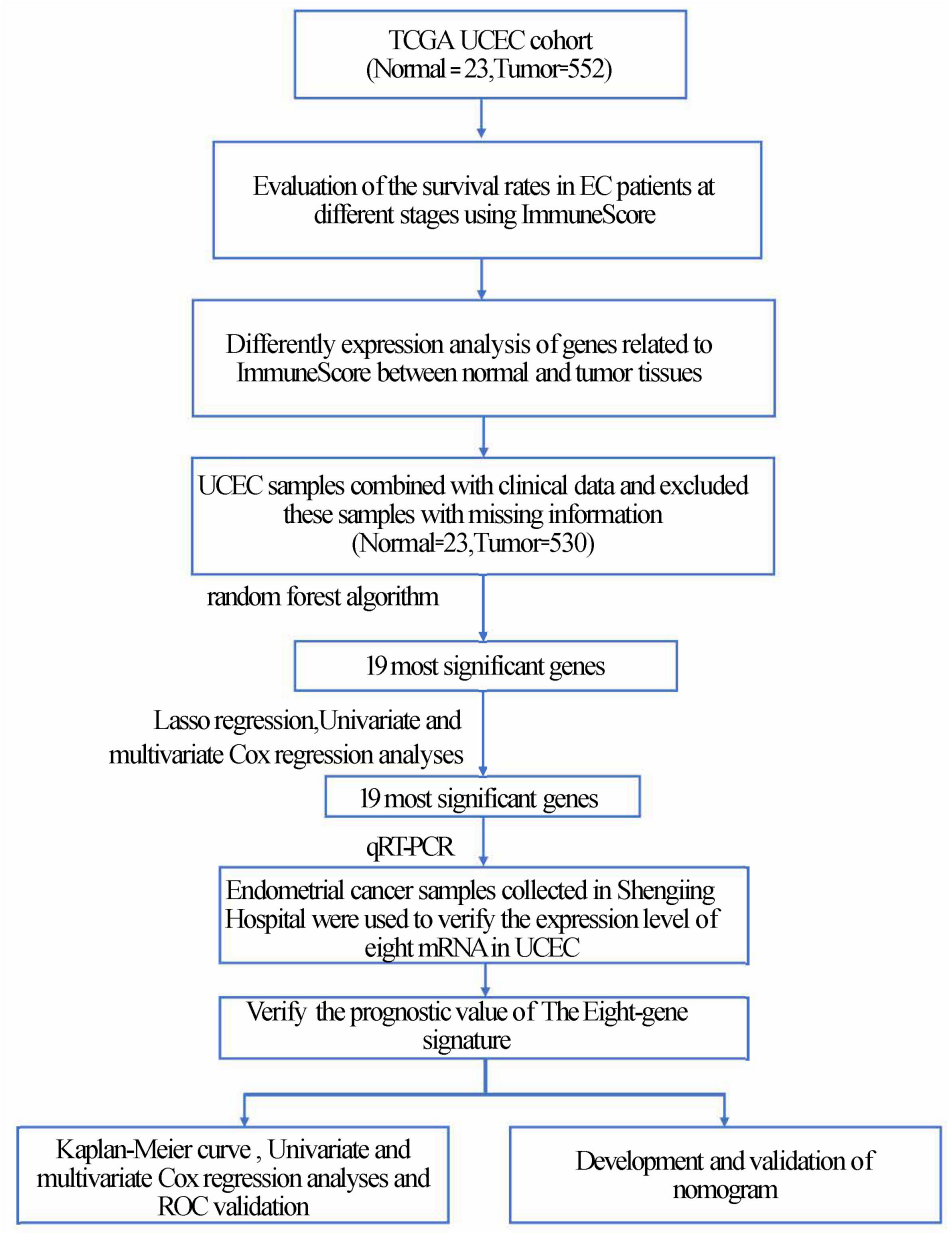
Flowchart of the article.

We applied Kaplan–Meier (K–M) survival curves and the log-rank means to evaluate the importance of the hazard parameters. We performed multivariate Cox regression and data lamination analyses to examine whether the risk parameters were individual clinical characteristics, containing age, stage, grade, radiation therapy, neoplasm cancer status, residual tumor, BMI, percentage of tumor invasion, new events, and peritoneal wash, which were used as covariates. Statistical significance was established at *P <* 0.05. Statistical analysis was performed using GraphPad Prism7 software (GraphPad, Inc., La Jolla, CA, USA) and SPSS software (version 20.0; SPSS, Inc., Chicago, IL, USA). The nomogram was constructed to evaluate prediction accuracy and recognition ability. The ROC curve (area under the curve [AUC]) was further applied to evaluate the discriminative ability of the nomogram (17, 18)(**Figure 1**).

## 3 Results

### 3.1 Evaluation of survival rates in EC patients at different stages using ImmuneScore

**Figure 2:**
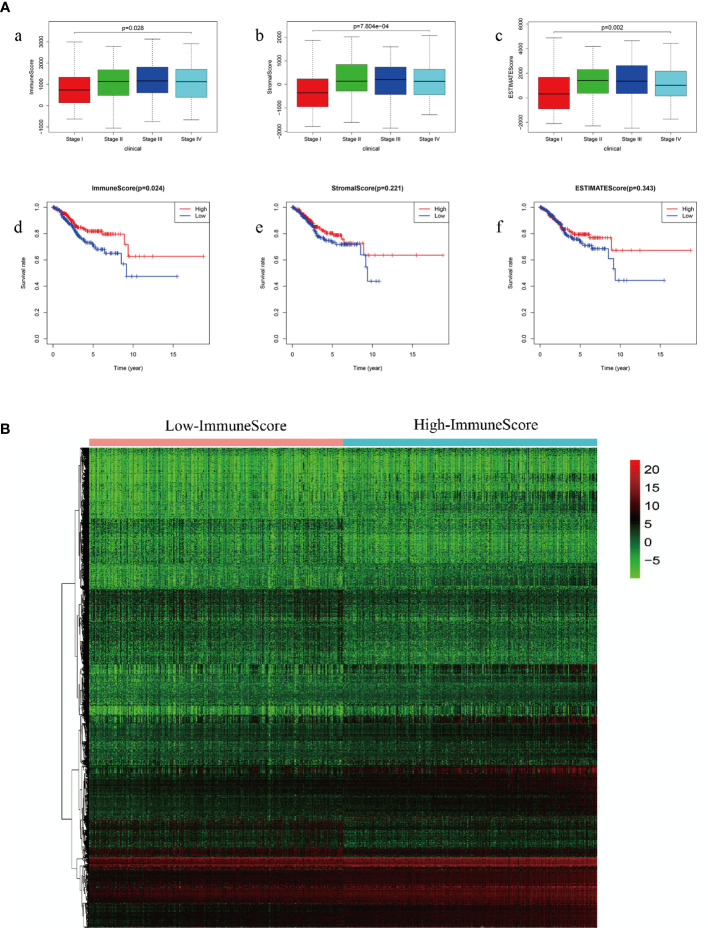
**(A)** Comparison of survival rates of ImmuneScore, StromalScore and ESTIMATEScore in different stages of endometrial carcinoma (a: ImmuneScore in different stages of endometrial carcinoma; b: StromalScore in different stages of endometrial carcinoma; c: ESTIMATEScore in different stages of endometrial carcinoma; d: comparison of survival rates of ImmuneScore; e: comparison of survival rates of StromalScore; f: comparison of survival rates of ESTIMATEScore) **(B)** ImmuneScore screens high-group and low-group differential genes.

We obtained the clinical characteristics of 530 patients with EC, along with the related 24,991 mRNA expression datasets from the TCGA database. Kaplan-Meier survival analysis was performed on ImmuneScore, StromalScore and ESTIMATEScore after they were generated. A higher ImmuneScore or StromalScore indicated a greater proportion of immune or stromal components in the TME. ESTIMATEScore was the sum of ImmuneScore and StromalScore, which represented tumor purity and represented the comprehensive ratio of the two components in TME. As shown in **Figure 2A**, the proportion of immune components was positively correlated with OS, while StromalScore and ESTIMATEScore were not significantly correlated with OS. These results implied that the immune components of TME were more suitable to indicate the prognosis of EC patients. According to the median ImmuneScore, 530 EC specimens were assigned to high and low-risk groups (**Figure 2B**).

### 3.2 Identification of mRNAs associated with survival

**Figure 3:**
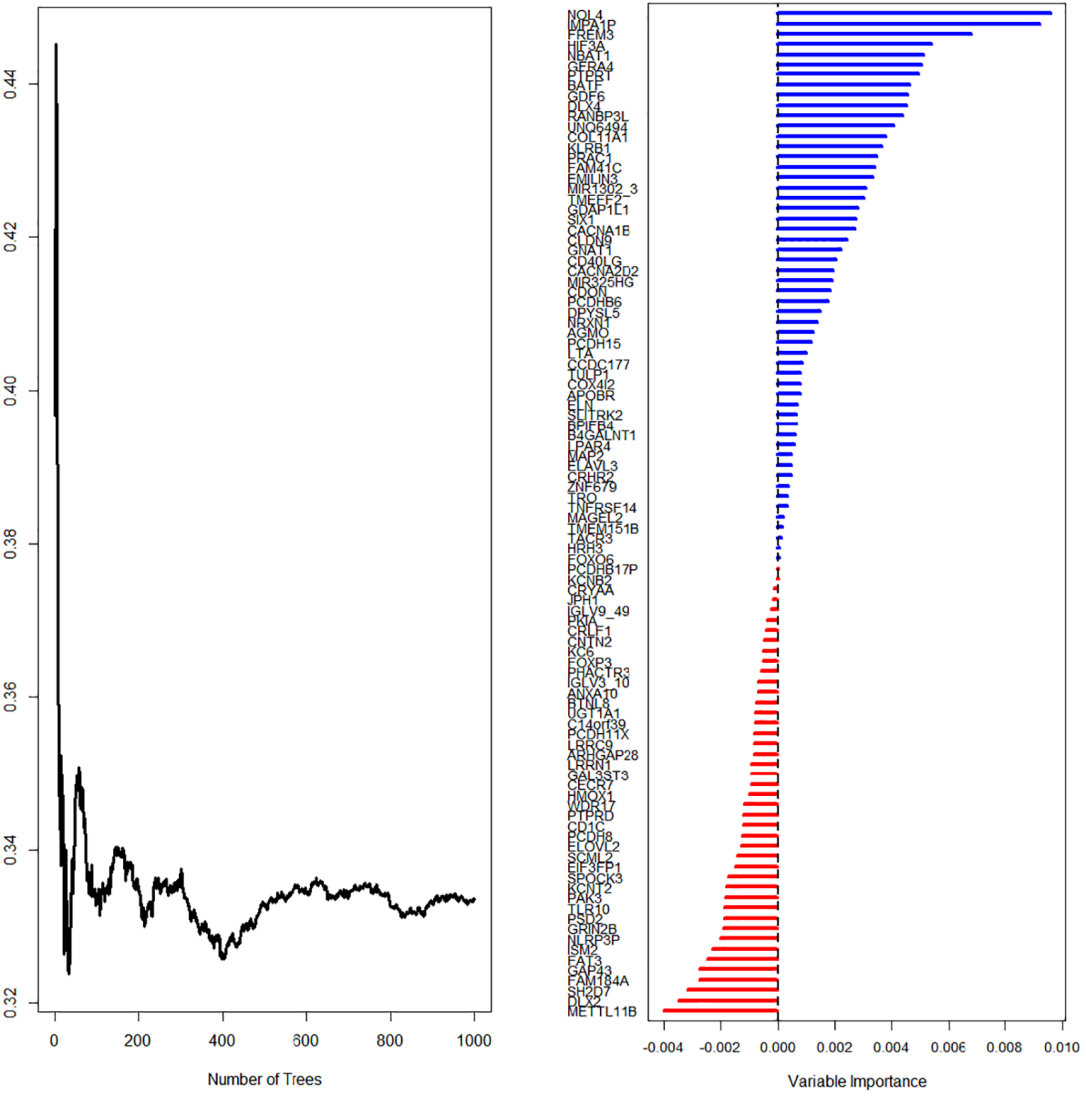
LASSO regression (iteration equal 1000) screening for survival-related mRNAs.

First, we screened out the differentially expressed ImmuneScore-related genes using the random forest algorithm, and obtained 19 best genes (*P <* 0.05) (**Figure 3**). Then, The least absolute shrinkage and selection operator (LASSO) regression (iteration equal 1000), univariate and multivariate Cox regression analyses were used to further verify the correlation between the 19 mRNA expression profiles and patient survival rates, and to clarify the best mRNA associations using the stepwise cleaning method. As shown in **Table 2**, eight mRNAs (*NBAT1*, *GFRA4*, *PTPRT*, *DLX4*, *RANBP3L*, *UNQ6494*, *KLRB1*, and *PRAC1*) were verified. After filtering, six mRNAs were classified as risky (*NBAT1*, *GFRA4*, *PTPRT*, *DLX4*, *RANBP3L*, and *PRAC1*) with HR>1 related to poorer survival, and two as protective types (*UNQ6494* and *KLRB1*) with HR<1 related to better survival (**Table 2**).

**Table 2:** Details of 8 prognostic mRNAs significantly related to survival in patients with endometrial cancer.

mRNA	B (Cox)	HR	P
NBAT1	0.0127	1.0127	0.0004
GFRA4	0.0005	1.0005	0.0003
PTPRT	0.0003	1.0003	0.0158
DLX4	0.0014	1.0014	0.0001
RANBP3L	0.0108	1.0109	0.0051
UNQ6494	-0.0296	0.9709	0.1588
KLRB1	-0.0035	0.9965	0.1903
PRAC1	0.0058	1.0058	0.0692

### 3.3 Verification of TCGA expression using qRT-PCR

**Figure 4:**
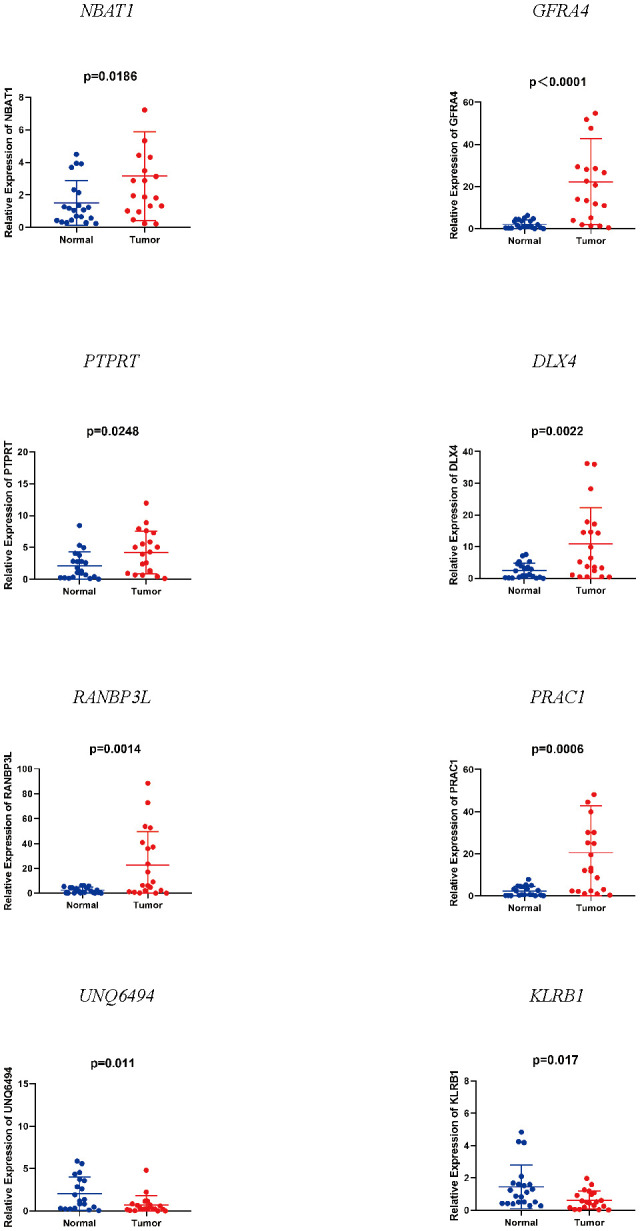
Expression of eight mRNAs in endometrial cancer tissues and normal tissues.

We detected notable changes in the expressions of eight mRNAs from 20 EC tissues and 20 normal endometrial tissues by qRT-PCR. We performed the unpaired t-test to assess the variation in mRNA expression of the two groups. The results showed that NBAT1, GFRA4, PTPRT, DLX4, RANBP3L, and PRAC1 were up-regulated, whereas UNQ6494 and KLRB1 were down-regulated in EC tissues as compared with that in normal endometrial tissue (**Figure 4**). The changes verified by qRT-PCR in mRNA expression levels in the 20 patients with EC were identical to the predicted changes obtained from bioinformatics analysis, confirming the significance and accuracy of these results.

### 3.4 Construction of an eight-mRNA signature to forecast patient prognosis

**Figure 5:**
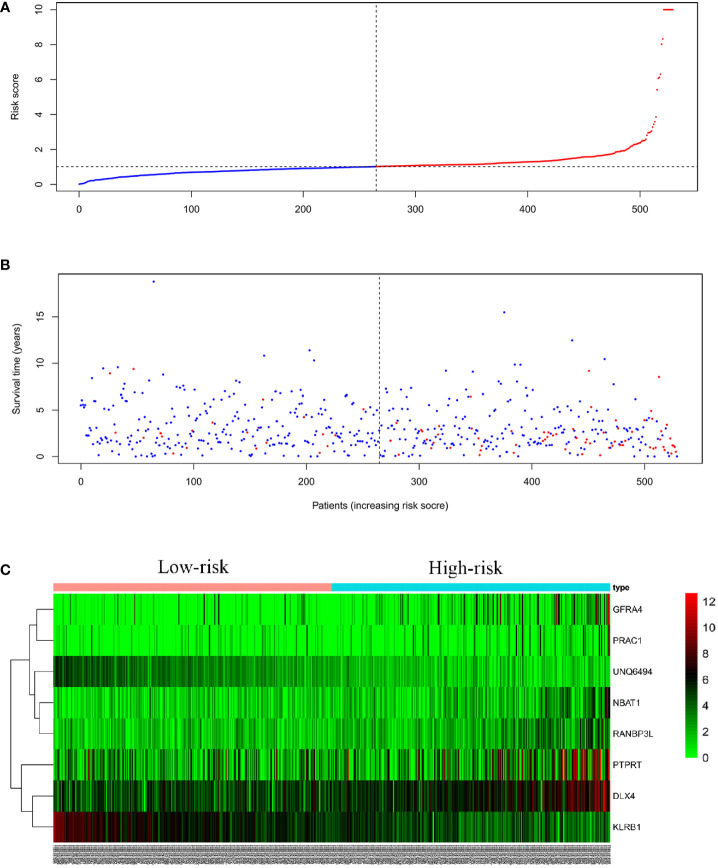
Eight mRNA signatures related to riskScore predict OS in endometrial cancer patients **(A)** Distribution of mRNA riskScore for each patient (the red line represents high risk, the blue line represents low risk) **(B)** Survival time (years) of EC patients in ascending order of riskScore (the red dots represent dead patients, the blue dots represent surviving patients) **(C)** A heatmap of eight genes expression profile.

We linearly integrated the expression values of the selected genes and the values of these genes weighted by the coefficients obtained from the multivariate Cox regression analysis. We derived the following formula to evaluate the prognosis: Risk parameters = 0.0127 × expression of *NBAT1* + 0.0005 × expression of *GFRA4* + 0.0003 × expression of *PTPRT* + 0.0014 × expression of *DLX4* + 0.0108 × expression of *RANBP3L* + 0.0058 × expression of *PRAC1* − 0.0296 × expression of *UNQ6494* − 0.0035 × expression of *KLRB1*. We computed the parameters in all patients and assigned hazard parameters to them. We ranked the patients in ascending order according to the parameters and used the median to divide them into high- and low-risk subgroups (**Figure 5A**). The life span of each patient is shown in **Figure 5B**. The mortality rate in patients in the high-risk parameter group was higher, whereas the survival rate in patients in the low-risk parameter group was better. Furthermore, the heat map shows the expression profiles of eight mRNAs (**Figure 5C**). The expression levels of risky-type mRNAs (*NBAT1*, *GFRA4*, *PTPRT*, *DLX4*, *RANBP3L*, and *PRAC1*) were higher in the high-risk group than in the low-risk group. In comparison, The expression levels of protective-type mRNA (*UNQ6494* and *KLRB1*) were lower in the high-risk group than in the low-risk group.

### 3.5 Risk parameters obtained from the eight-mRNA signature as single-handed prognostic indicators

We compared the prognostic significance of the risk parameters and clinicopathological parameters using univariate and multivariate analyses (**Table 1**). Specimens with good clinical data were chosen for the analysis. The median age of the 528 patients with EC was 66 years. The median BMI of the 500 patients with EC was 28. Of the 530 patients, 60 (11.3%) had new events during the follow-up period and 47 (8.9%) were treated with radiation therapy. Of the 440 patients, 75 (17.0%) had residual tumors. Of the 494 patients, 76 (15.4%) had a tumor in neoplasm cancer status. Of the 457 patients, 43 (9.4%) had tumor invasion. Of the 401 patients, 57 (14.2%) had a tumor in peritoneal wash. Of the 530 patients, 216 (40.8%) had grade 1-2 tumors, and the remaining 314 (59.2%) had grade 3-4 tumors. In addition, among these patients, 381 (71.9%) were in stage I-II, and 149 (28.1%) were in stage III–IV. Based on the above data, we used the risk score, age, neoplasm cancer status, stage, grade, residual tumor, new tumor events, and percent tumor invasion as single-handed prognostic symbols, because these factors showed noticeable discrepancies in univariate and multivariate analyses (**Table 3**). Notably, the risk score showed a significant prognostic value (*P <* 0.05) (HR = 1.054).

**Table 3:** Univariable and multivariable analyses for each clinical feature.

Clinical feature	Number	Univariate analysis HR 95%CL of HR P-value	Multivariate analysis HR 95%CL of HR P-value
RiskScore (High‐risk/Low‐risk)	274/256	1.070 1.052-1.089 0.00	1.054 1.029-1.079 0.00
Age (≥ 66/< 66)	262/266	1.667 1.080-2.571 0.02	1.309 0.775-2.209 0.31
Stage (I-II/III–IV)	381/149	2.013 1.662-2.438 0.00	1.404 1.055-1.869 0.02
Grade (G1-2/G3–4)	216/314	2.593 1.804-3.725 0.00	1.684 1.092-2.596 0.02
Residual tumor (yes/no)	75/365	2.888 1.784-4.674 0.00	0.788 0.415-1.496 0.47
New tumor event (yes/no)	60/470	4.451 2.863-6.921 0.00	2.010 1.120-3.606 0.02
Neoplasm cancer status (with tumor/tumor free)	76/418	8.860 5.704-13.761 0.00	3.079 1.514-6.262 0.00
Percent tumor invasion (yes/no)	43/414	1.008 1.001-1.015 0.02	1.006 0.998-1.015 0.12

### 3.6 K–M survival estimation to verify eight-mRNA signatures for prognosis prediction

**Figure 6:**
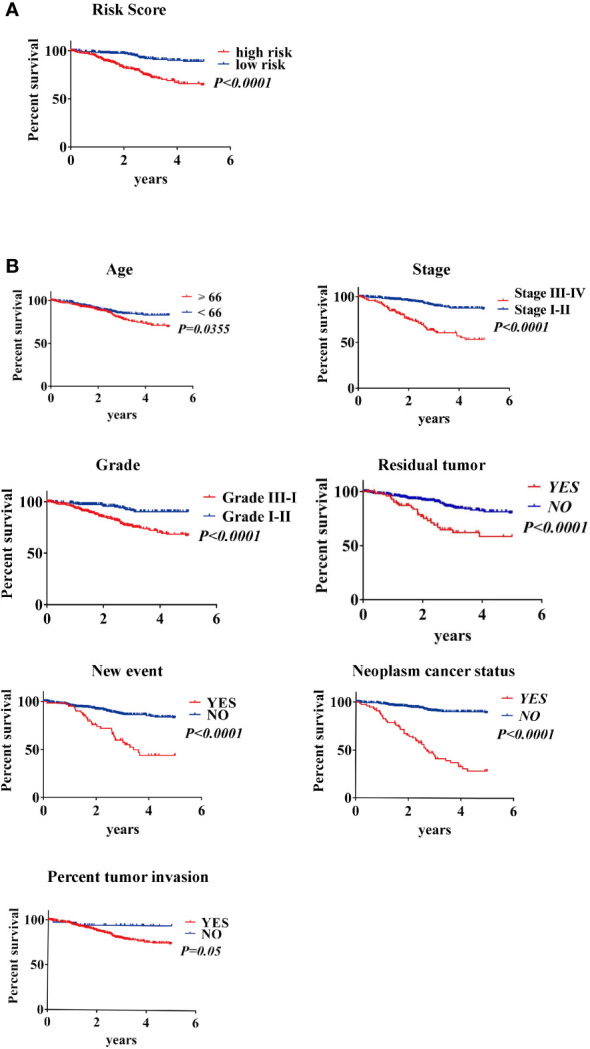
Kaplan–Meier survival analysis of EC patients in the TCGA data set **(A)** K–M survival curve of high/low-risk EC patients **(B)** Clinical features, including age, stage, grade, residual tumor, new event, neoplasm cancer status, and percent tumor invasion, predict patient survival.

**Figure 7:**
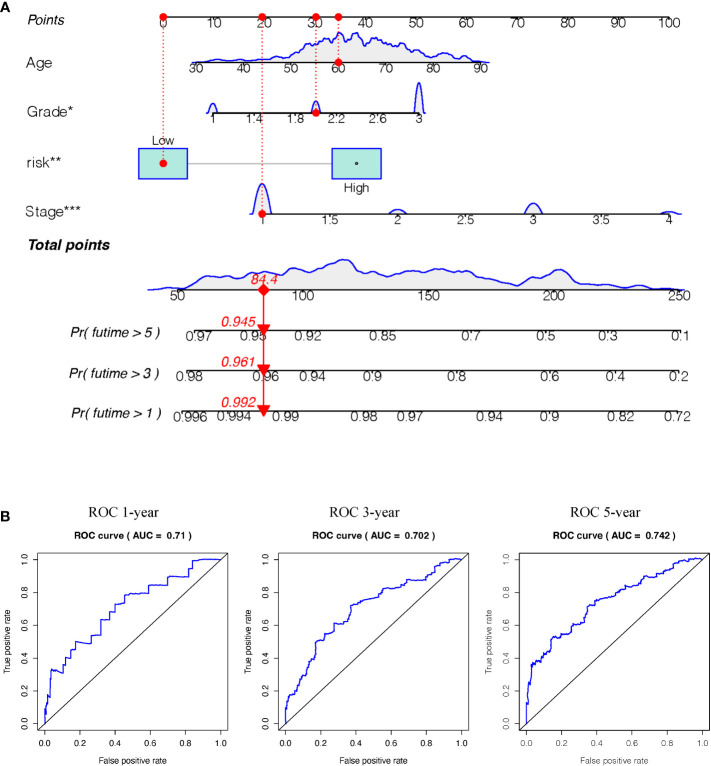
**(A)** Nomogram (for OS) that integrated the clinicopathologic risk factors. To calculate the probability of status, the points identified on the scale for all the variables are summed up and a vertical line was drawn from the total points scale to the probability scale.(Stage: 1 means FIGO stage1, 2means FIGO stage2, 3 means FIGO stage3, 4 means FIGO stage4. Grade: 1 means well-differentiated, 2 means moderately differentiated, 3 means poorly differentiated) **(B)** ROC curves showing the predictive accuracy (1-, 3-, 5-year AUC) of the nomogram for OS in testing and validation cohorts. (*P<0.05, **P<0.01, ***P<0.001).

Survival estimates of K-M and logarithmic tests showed that patients in the high-risk group had a poorer prognosis (**Figure 6A**). Univariate Cox regression analysis of OS identified some clinicopathological parameters that could predict EC survival, such as age, grade, stage, residual tumor, new event, neoplasm cancer status, BMI, radiation therapy, peritoneal wash, and percentage of tumor invasion. We then used K–M survival assessment to verify the conclusions obtained. These conclusions gave identical results for patients above 66 years old, with G3-4 tumors, stage III–IV disease, neoplasm cancer status, tumor recurrence (new event), tumor invasion, and with poor prognosis of residual tumor (**Figure 6B**). Concurrently, based on the results of the multivariate model, a nomogram was constructed that combined clinical parameters. Based on established prognostic factors, it could provide a clinically useful quantitative method for predicting the probability of survival at 1, 3, and 5 years in patients with EC (**Figure 7A**). The analysis of the ROC curve showed the prediction accuracy of the nomogram in the test and validation cohorts (1-, 3-, and 5-year AUC) (**Figure 7B**). These results proved the accuracy of the analysis.

### 3.7 Cellular functional experimental validation of NBAT1

**Figure 8:**
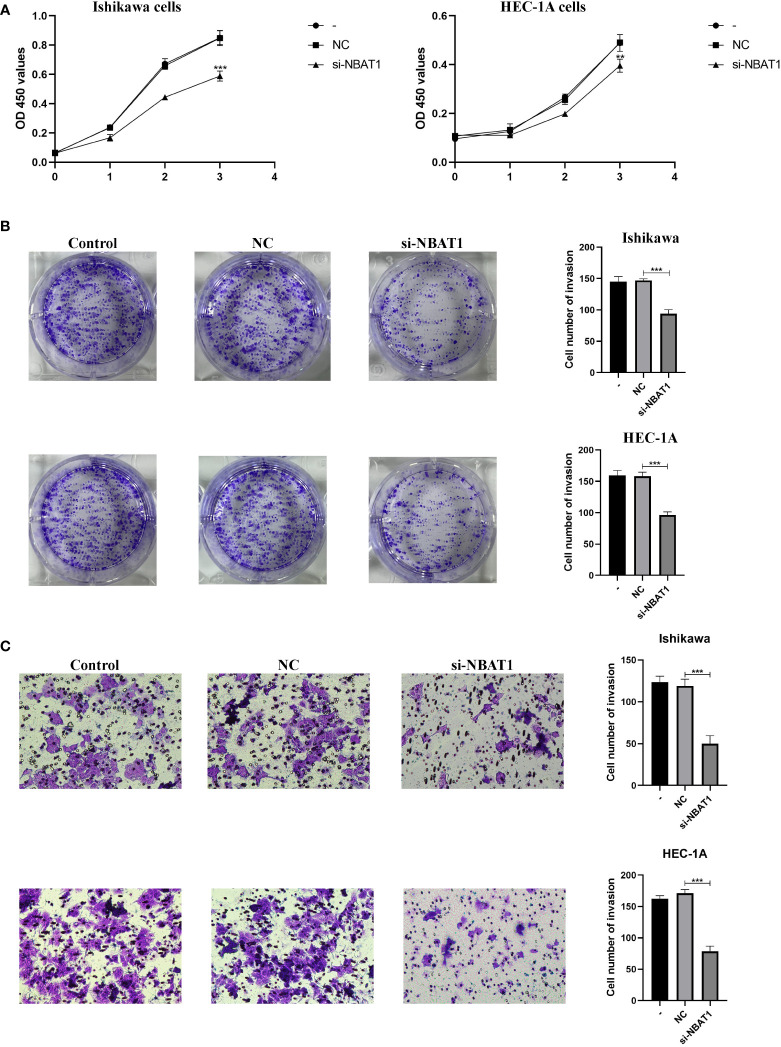
NBAT1 regulates the biological behavior of Ishikawa cell line and HEC-1A cell line. **(A, B)** CCK-8 and colony formation assays were used to assess the proliferative effect of NBAT1 **(C)** Effect of NBAT1 on invasion assessed using the Transwell assay. (**P<0.01, ***P<0.001).

To further verify the roles of the 8 genes in EC, we selected NBAT1, the most representative gene among the 8 genes, according to COX and HR values. The expression of NBAT1 was knocked down in Ishikawa cells and HEC-1A cells, and the knockdown effect of NBAT1 was verified by PCR in **Supplementary Table S4**. Knockdown of NBAT1 inhibited the proliferation (**Figures 8A, B** **)** and invasion (**Figure 8C** **)** of ECs. The accuracy of these results was further confirmed by cellular functional experiments, which verified that the expression of NBAT1 was identical to the predicted changes obtained from the bioinformatics analysis.

## 4 Discussion

Accurate risk stratification and long-term prognostic prediction are essential for the correct selection of treatment modes for patients with EC. Integrating multiple independent prognostic variables into a single formula can significantly improve the prognostic ability (19). An increasing number of studies have shown that genes can influence tumor progression by regulating the cell cycle, thereby providing candidates for targeted therapy. Therefore, the identification of prognostic EC biomarkers is essential to improve preoperative and postoperative risk assessments and guide treatment decisions. The stratification system for EC is based on a few molecules, mainly clinical and pathological parameters, but this system remains inaccurate. In this study, we constructed and verified ImmuneScore to compare survival rates in patients with EC at different stages to improve their prognosis prediction.

Recently, immune profiling studies have taken a leading position in cancer research. Several studies based on ImmuneScores have been published to describe the immune landscape and provide independent prognostic models for the survival of patients with several types of solid tumors, including gastric and liver cancers (19–21). In addition, previous data have also shown that specific immune cells were closely related to treatment response to therapies (such as chemotherapy and immune-modulating therapies) (22). However, previous studies have established many molecular signatures (including genes, microRNAs, lncRNAs, and epigenetic biomarkers) to predict long-term survival in cancer patients (20, 23, 24). These features have not been widely used in clinical practice due to variability in gene sequencing measurements, inconsistent testing platforms, and the requirement for specialized analysis. In this study, we used ImmuneScore combined with the edgeR algorithm and the LASSO model, as well as the nomogram and ROC curve verification, which might be widely used in clinical practice.

The molecular classification of TCGA is expected to provide additional prognostic information; therefore, it is expected to improve the ESMO-ESGO-ESTRO risk stratification system. Studies conducted in large cohorts of patients, especially those conducted using TCGA (other cohorts), Vancouver, and PORTEC groups (25–29) have verified their prognostic relevance and pointed out that they will benefit from this classification system. In particular, it was reported that 7% of patients diagnosed with cancer with good prognosis (EC Grade 1) but with copy number polymer diagnosis were now classified into the poor prognosis group (27). In contrast, all patients with POLE-hypermutation tumors (6 –13% of all EC tumors) are now considered good prognostic tumors regardless of the status of other prognostic factors (such as histological grade or FIGO stage).

The fast progress of high-throughput gene sequencing technique has laid the basis for large-scale biological data study (30). All the genomic data is screened from a single specimen to distinguish fresh diagnostic, prognostic, or pharmacological biomarkers (31). The combination of biomarkers provides discriminative power higher than molecular tests based on a single marker. Furthermore, as observed in a study by Yang et al., integrating molecular biomarkers with clinicopathological characteristics may be the easiest strategy to develop more sensitive and specific tests (32). In this study, we used ImmuneScore to determine the difference in survival rates in patients with EC at different stages, combined with edgeR to screen out the differential genes, and obtained the best gene combination using the LASSO model.

In recent studies, new prognostic markers of gene expression levels or mutations have been constructed by applying microarray and RNA sequencing data. The Cox proportional hazards regression model was applied to identify and validate these markers (33, 34). In this study, we identified 19 gene combinations using the LASSO model. We chose advanced features to screen for genes associated with sufferer survival forecast, instead of widespread exploration. Univariate and multivariate Cox regression analyses were used to clarify the prognostic significance of these eight-gene combinations in patients with EC. Compared to currently known prognostic evaluation indicators, this selected hazard contour may be a better targeted approach and a more powerful prognostic evaluation to predict positive clinical results.

Tumor immunotherapy is now receiving more and more attention and is recognized as a new and effective method for cancer treatment, and good clinical responses have been observed in some relapsed and refractory cases (35–37). Immune checkpoint inhibitors (ICIs), cancer vaccines, adoptive cell transfer (ACT), and lymphocyte-promoting cytokines are the main immunotherapy approaches, while immunotherapy targeting different EC subtypes (especially POLE and MSI-H) has also gradually attracted attention (38). As endometrial cancer pathogenesis is further elucidated, more and more evidence shows that a large number of immune cells and cytokines can be seen in endometrial cancer tissue, which can stimulate endogenous antitumor immune responses. Compared with other gynecologic malignancies, endometrial cancer is most likely to benefit from immunotherapy (39–41). Certain immune environment signature parameters are often associated with ImmuneScores and can assess prognosis in other cancer types. Therefore, these characteristic parameters can be effective prognostic factors before and after treatment and can be used as predictive immune parameters in planning appropriate interventional treatment (42). The ImmuneScore provides a reliable estimate for predicting the recurrence risk of EC patients.

In this study, bioinformatic methods were applied to discover the characteristics and clinical significance of mRNA hazard factors and to explore a new method to discover potential prognostic markers. We applied the EC dataset in TCGA to screen concerning genes through ImmuneScore, compared the data of different stages using EC tissue specimen data, and classified them using high- and low-risk ImmuneScores. For patients with low-risk parameters, K-M survival estimates showed a beneficial prognosis. We demonstrated the effects of 8 genes on EC prognosis and survival by qRT-PCR experiments and cellular experiments. At the same time, the discovery and calculation of hazard parameters for EC patients has great clinical significance. Due to the lack of data on metastasis and recurrence in EC patients in the TCGA database, one limitation of our study is that we could only apply the OS parameter to evaluate the prognosis in these patients. A second limitation was that all specimens were retrospectively obtained from the TCGA database. Therefore, our results need to be verified in a larger prospective cohort study. Furthermore, in the stratified analysis, the risk parameters in all subgroups could predict the prognosis in EC patients, except for the subgroup aged <66 years. The cause of this discrepancy remains unclear and needs further in-depth research.

Realizing the clinical implementation of biomarkers is another important discussion. Once verified, the biomarker should ideally be transferred to a standardized, economical, simple, and fast analysis platform, and should be prospectively verified in accordance with all regulatory requirements to become an *in vitro* diagnostic test. However, no relevant genetic markers have been established to predict the prognosis in patients with EC. Using bioinformatics methods, we clarified the genetic characteristics related to ImmuneScore (*NBAT1*, *GFRA4*, *PTPRT*, *DLX4*, *RANBP3L*, *UNQ6494*, *KLRB1*, and *PRAC1*), and proved their prognostic value in EC. At the same time, we also made some comparisons with existing literature, such as Jiang et al. (43) and Liu et al. (44). The method we used showed better performance and stability, and ImmuneScore could be used for the prognosis of EC patients, providing reliable estimates, which highlights the good predictive performance of our eight-gene signature.

Recent studies have shown that TME also played a role in tumor occurrence and progression. Discovering latent therapeutic targets that can help shape TME and accelerate the transition of TME from tumor-friendly to tumor-suppressed state has great benefits. Many studies have shown the significance of the immune microenvironment in tumorigenesis. In particular, the rate of immune and stromal compositions in TME is closely related to tumor progression, such as invasion and metastasis (45). These consequences highlight the importance of discovering the interplay between tumor cells and immune cells, which provides new insights for the development of more valid therapy options. The type of immunity may have a significant impact on individualized follow-up and adapted treatment decisions after surgery.

## 5 Conclusion

We obtained an eight-gene signature risk profile that can predict the prognosis in patients with EC using ImmuneScore, and higher risk parameters were associated with a poor prognosis. This signature can be used as a classification tool in clinical practice. These findings provide the strategy for accurate identification of patients with EC with a poor prognosis.

## Data availability statement

The datasets presented in this study can be found in online repositories. The names of the repository/repositories and accession number(s) can be found in the article/**Supplementary Material**.

## Ethics statement

All patients gave informed consent. This study was approved by the Ethics Committee of Shengjing Hospital affiliated with the China Medical University.

## Author contributions

JG participated in the design, methodology, data interpretation, and analysis for the work; carried out the statistical analyses; and drafted the manuscript. ZW participated in the methodology, data interpretation, and analysis of the work. BW carried out the statistical analyses. XM designed the study; participated in data interpretation, analysis for the work, and methodology. All authors read and approved the final manuscript.

## Supplementary material

Supplementary Figure 1
**(A)**Decision Curve Analysis (DCA) of the model (The red line represents the curve of Riskscore) **(B)** Calibration Curve Analysis(CCA) of the model.

Supplementary Figure 2 TIMER to assess sample immunity scores.

Supplementary Figure 3 Relationship between 8 mRNAs and clinicopathologic parameters of endometrial carcinoma.
