# Supplementary figures and images for: ImmuneScore of eight-gene signature predicts prognosis and survival in patients with endometrial cancer
Source: Front Oncol. 2023 Mar 3;13:1097015. doi: 10.3389/fonc.2023.1097015 (PMC10020521; doi:10.3389/fonc.2023.1097015)

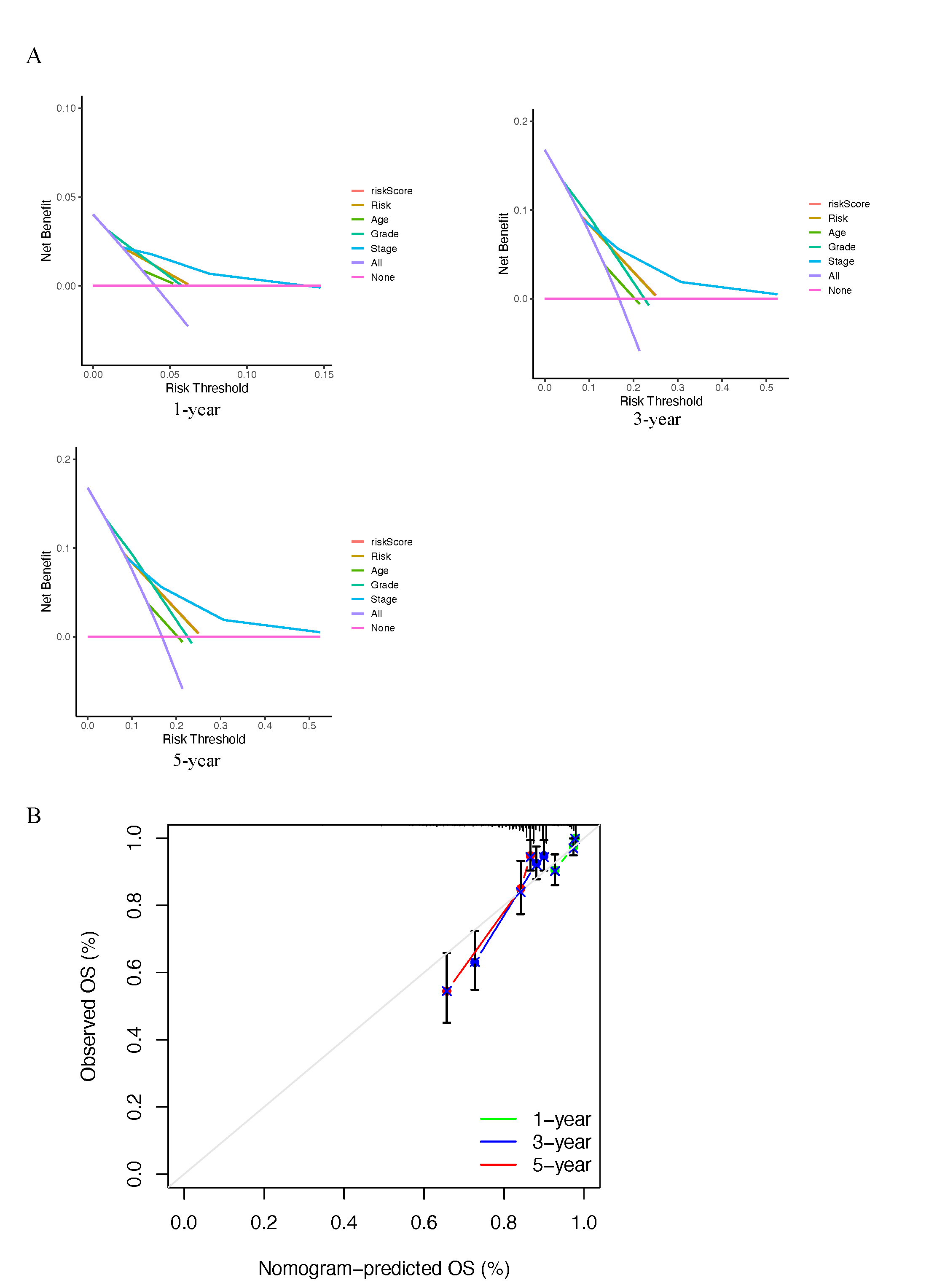

Supplement: Supplementary Figure 1 — (A)Decision Curve Analysis (DCA) of the model (The red line represents the curve of Riskscore) (B) Calibration Curve Analysis(CCA) of the model. [file Image_1.tiff]

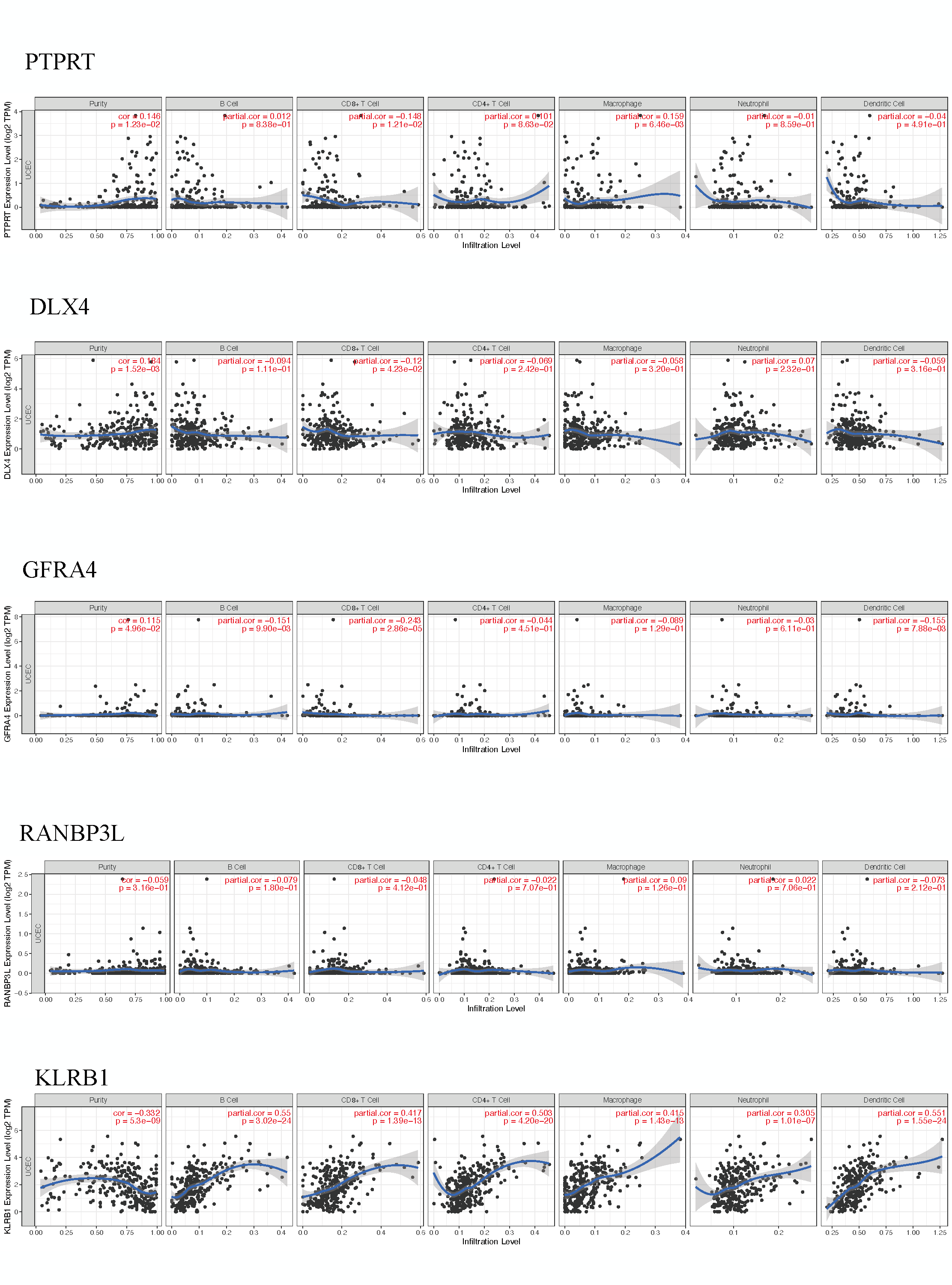

Supplement: Supplementary Figure 2 — TIMER to assess sample immunity scores. [file Image_2.tiff]

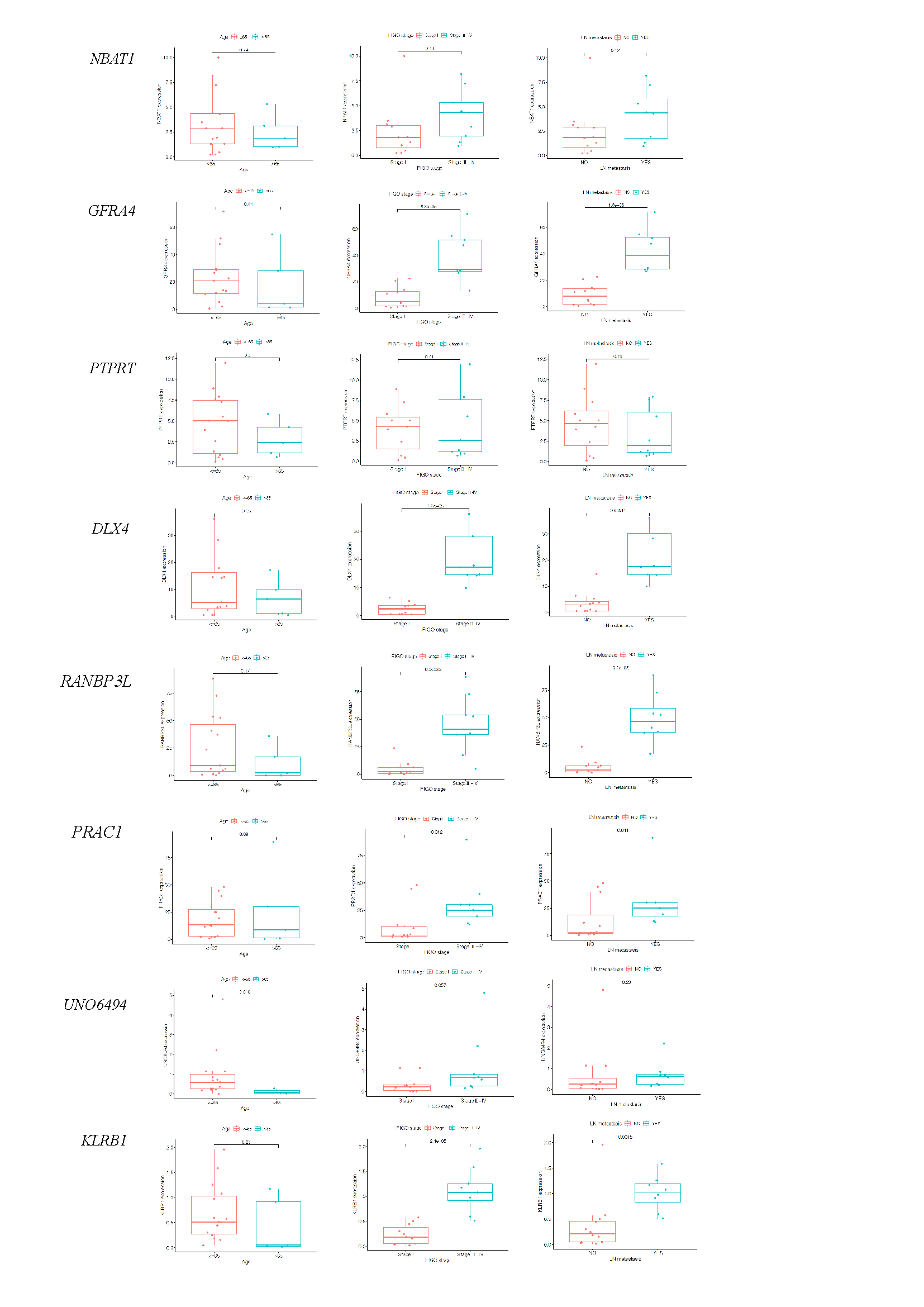

Supplement: Supplementary Figure 3 — Relationship between 8 mRNAs and clinicopathologic parameters of endometrial carcinoma. [file Image_3.tiff]

**Supplementary Table S4: siRNA knockdown efficiency results for NBAT1**

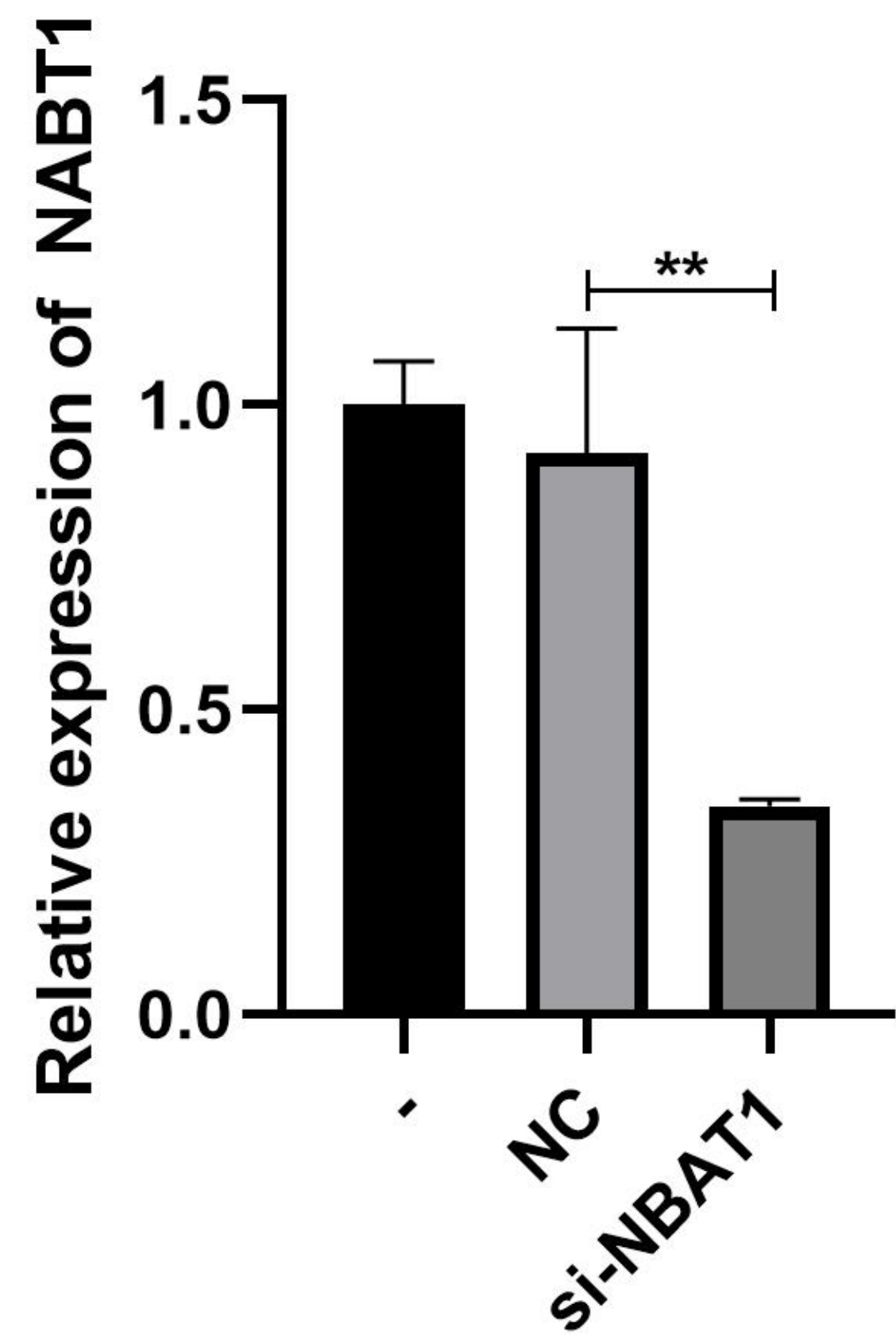

Supplement: Supplementary file 7 [file Table_4.pdf]
