# Supplementary material for: ImmuneScore of eight-gene signature predicts prognosis and survival in patients with endometrial cancer
Source: Front Oncol. 2023 Mar 3;13:1097015. doi: 10.3389/fonc.2023.1097015 (PMC10020521; doi:10.3389/fonc.2023.1097015)
Supplement: Supplementary file 4 [file Table_1.pdf]

Clinicopathological data of 20 patients with endometrial cancer

Clinical parameters	N
Age	
<60	15
≥60	5
Grade	
G1-2	17
G3-4	3
Differentiation	
Low + Middle	9
High	11
Invasion depth	
Superficial	8
Deep	12
Lymphatic metastasis	
No	18
Yes	2
Vascular invasion	
No	20
Yes	0
Distal metastasis	
No	20
Yes	0
